# An Increased Presence of Male Personalities in Dissociative Identity Disorder after Initiating Testosterone Therapy

**DOI:** 10.1155/2020/8839984

**Published:** 2020-10-05

**Authors:** Monique Mun, Mohan Gautam, Renee Maan, Bassem Krayem

**Affiliations:** Henry Ford Health System, Department of Psychiatry, 1 Ford Place, 1C-09 Detroit, Michigan, USA 48202

## Abstract

Patients with gender dysphoria (GD) report significant dissociative symptoms and are found to have a high prevalence of a dissociative disorder of any kind. When GD patients elect to undergo cross-sex hormone therapy, there is a significant reduction in dissociative symptoms. However, to the best of our knowledge, there are no known case reports that describe an alteration of personalities in dissociative identity disorder after initiating cross-sex hormone therapy. Thus, we present a case of a 20-year-old transgender male with GD, whom after initiating cross-sex hormone therapy with testosterone experienced an increased presence of his existing male personalities.

## 1. Introduction

Gender dysphoria (GD) is defined in the DSM-V as significant distress due to an incongruence between one's experienced or expressed gender and one's assigned gender. It has a prevalence ranging from 0.5 to 1.3% in the United States [1]. Patients with GD report significant dissociative symptoms, such as depersonalization/derealization, absorption/imaginativeness, and conversion [2]. Thus, patients with GD have a high prevalence of a dissociative disorder of any kind (29.6%) [3]. When GD patients elect to undergo cross-sex hormone therapy, there is a significant reduction in dissociative symptoms [4]. However, to the best of our knowledge, there are no case reports that describe an alteration of personalities in dissociative identity disorder after initiating cross-sex hormone therapy. Thus, we present a case of a 20-year-old transgender male with GD, whom after initiating cross-sex hormone therapy with testosterone, experienced an increased presence of his existing male personalities.

## 2. Case

A 20-year-old transgender male with GD, undergoing cross-sex hormone therapy with testosterone, with past psychiatric history of dissociative identity disorder (DID), PTSD, and depression, was admitted to our inpatient psychiatric hospital after endorsing suicidal ideations and homicidal ideations towards others. This is context to significant distress from recently switching to one of his personalities. Family history was positive for depression in his mother and sister. Labs were unremarkable, except for elevated hemoglobin (16.7 g/dL), elevated hematocrit (48.7%), and elevated RBC count (6.12 M/*μ*L). Urine drug screen was positive for cocaine, amphetamine, and benzodiazepine. The patient did not recall use of these substances, citing they were done as his most recent personality, Dexter.

The patient has a history of sexual abuse by his mother's boyfriend from when he was 3 years old. He was subsequently diagnosed with PTSD and depression in his early adolescence. Although he no longer endorsed having nightmares, he continued to have intrusive-type symptoms of PTSD and a negative emotional state of anger and fear. While he was seeking outpatient therapy for PTSD and depression, his therapist diagnosed him with DID. Although he was born as the female sex, he identified having 6 personalities, most of whom are the male gender.

The patient started testosterone therapy two years prior to presentation, which has alleviated his GD symptoms. However, since starting testosterone, he noticed an increased presence of two existing personalities, both of whom are male: (1) Dexter, a personality who is aggressive and hostile and has violent thoughts—he uses illicit drugs; and (2) Bad, a personality who is evil and destructive—he throws furniture when he is angry and even tried to assault his girlfriend.

## 3. Discussion

DID, formerly known as multiple personality disorder, falls under the category of dissociative disorders in the DSM-V, alongside dissociative amnesia, depersonalization/derealization disorder, other specified dissociative disorder, and other unspecified dissociative disorder [5]. Dissociative disorders have a prevalence of 5-10% in the general population [6]. The hallmark of dissociative disorders is “an interruption and/or discontinuity in the normal integration of consciousness, memory, self-identity and subjective identity, emotion, perception, body identity, motor control and behavior” [6]. There are various factors that may confound or exacerbate symptoms of dissociative disorders, such as drugs of abuse, comorbid diagnoses with dissociative features, such as PTSD, and utilization of hormone therapy.

### 3.1. Drugs of Abuse and Dissociation

Drugs of abuse have been known to induce symptoms of dissociation that resemble dissociative disorders. Although the patient denied consistent drug use, his UDS was positive for cocaine, amphetamine, and benzodiazepine. A study by Kloet D et al. revealed that participants who were administered cannabis (THC 300 *μ*g/kg), MDMA (single doses of 25, 50 or 100 mg), or cocaine (HCl 300 mg) all had a significant increase in dissociative symptoms compared to placebo, as measured by the Clinician-Administered Dissociative States Scale (CADSS) [7]. Specifically, the most profound effects were found with cannabis and MDMA; the effect of MDMA on dissociative symptoms was dose-dependent [7]. Similar to cocaine, stimulants such as dexamphetamine and methylphenidate can also increase rates of dissociation and psychedelic state [8]. Benzodiazepines are used with caution in patients with dissociative disorders due to its potential to exacerbate symptoms of dissociation [9].

### 3.2. sPTSD and Dissociation

There is considerable overlap between DID and PTSD. They share a common risk factor of childhood trauma. The DSM-5 added a dissociative subtype to PTSD to capture patients with PTSD with a predominance of depersonalization/derealization symptoms and emotional detachment [5]. The prevalence of those with PTSD dissociative type and any type of dissociative disorder was as high as 54% [10]. Research has established a difference in physiologic and neural response to traumatic memories between dissociative type PTSD and those with nondissociative PTSD [11]. However, distinguishing between PTSD dissociative subtype and dissociative disorders relies on a structured clinical interview.

### 3.3. Testosterone and Dissociation

The psychiatric effects of androgens on mood are well-studied. Androgens are known to be associated with an increase in euphoria and energy but also irritability, violence, and hostility [12]. Specifically, testosterone acts in the subcortical structures of the hypothalamus and amygdala, where aggression and emotions are localized. The prefrontal cortex interprets and regulates aggression and emotions. High levels of testosterone downregulate the prefrontal cortex, resulting in an increased expression of aggression [13]. However, a research gap remains regarding effects of testosterone on dissociative symptoms and recollection of traumatic events. It is possible that initiating testosterone altered his experience of trauma recollection. In other words, perhaps, the patient's more masculine alters are a reflection of increased testosterone in patient's utilization of dissociation to manage traumatic recollections/events. This would be relevant because LGBTQ-based discrimination increases overall traumatization which has relevant implications in exacerbation of dissociation and its lasting impacts [14]. This becomes especially pertinent for this patient as those with worse emotional dysregulation are associated with more severe dissociation phenomena [15].

### 3.4. Treatment Recommendations

Treatment recommendation for DID is intensive individual psychotherapy that focuses on recognizing and encouraging the cooperation between personalities and adapting new coping skills [6]. Although there is not one psychotherapy modality that is recommended, psychodynamic psychotherapy and hypnotic interventions have been frequently used [6]. We recommended that the patient continues psychotherapy with his outpatient therapist, whom he was seeing regularly and had built rapport with. Comorbid diagnoses should be addressed and treated to reduce risk of suicidality. The patient's comorbid diagnoses of PTSD and depression were treated with venlafaxine after failing two previous trials of SSRIs. Venlafaxine was titrated to 225 mg daily. Substance use rehab was offered to the patient; however, he declined, citing he does not use substances consistently.

## 4. Conclusion

In summary, the present case reveals a correlation of testosterone therapy with dissociative symptoms. Although the patient reported a reduction in distress of his physical body after initiation of testosterone therapy, he reported an increase in hostility and violence, as well as an increased predominance of existing male alters. One cannot rule out the possibility of confounding factors that may have exacerbated or altered the patient's dissociative symptoms, such as substance use and clinical overlap of PTSD. Yet, there still exists a scarcity of literature that describes the effects of testosterone on dissociative symptoms. Areas of further research may focus on effects of testosterone on the severity of dissociative symptoms, the frequency of switching personalities, and changes to the alters. Similarly, the effects of other neurochemical compounds, such as estrogen, on dissociative symptoms are another area of potential research.
